# Low-value practices in oncology contributing to financial toxicity

**DOI:** 10.3332/ecancer.2017.727

**Published:** 2017-03-16

**Authors:** Bishal Gyawali

**Affiliations:** Department of Hemato-Oncology, Nobel Hospital, Sinamangal, Kathmandu 21034, Nepal

**Keywords:** financial toxicity, cancer, value, cost saving, oncology

## Abstract

Financial toxicity of cancer treatment is now a well-recognised problem in cancer medicine leading to patient bankruptcy and even poor survival, including in high-income countries and countries with public health care systems. Many oncologists, despite acknowledging the severity of financial toxicity as a problem, resign the responsibility of reducing the costs of cancer treatment to the government, industry, and oncology societies. However, an oncologist can play an important role in reducing the costs of cancer treatment because all cancer treatment decisions are made between the oncologist and the patient. In this article, I point out a few examples of low value practices from various oncology disciplines that we oncologists can easily replace or abandon in our practice and contribute to lessening the financial toxicities to patients and society. As these examples suggest, reducing cost does not necessarily mean compromising efficacy. We should continuously keep looking for other similar cost-saving strategies in our practice.

Financial toxicity of cancer treatment is now a well-recognised problem in cancer medicine leading to patient bankruptcy and even poor survival [1, 2]. Interestingly, however, the impact of financial toxicity is now no longer limited to low- and middle-income countries or poor patients. Recent studies have highlighted that financial toxicity leads to detrimental outcomes in oncology care even in high-income countries and countries with public health care systems [3, 4]. Thus, financial toxicity has become of paramount importance in the recent cancer literature [5]. The reasons are obvious: modern cancer drugs are no longer affordable. In 2012, 12 of 13 new anticancer drugs approved were priced over $100,000 a year in the United States [6]. Today, a full-course treatment with drugs such as pembrolizumab costs in excess of a million dollars [7].

The literature abounds with studies exploring the causes and consequences associated with the skyrocketing cost of anticancer drugs [8–11]. While the discussion and awareness of financial toxicity is the first and very important step, there can be no real benefits to patients and society without taking the appropriate steps to address these unique toxicities. However, many oncologists, despite acknowledging the severity of financial toxicity as a problem, resign the responsibility of reducing the costs of cancer treatment to the government, industry, and oncology societies. Indeed, the responsibility of reducing the costs of cancer treatment primarily lies in the coordination between the government and the industry. Oncology societies also do have an important role to play by raising awareness of these issues and lobbying to reduce the costs. The ASCO value framework [12] and the ESMO-Magnitude of Clinical Benefit Scale (MCBS) [13] are praiseworthy contributions to that end. However, as all cancer treatment decisions happen between the oncologist and the patient, the scope for reducing cancer treatment costs lies greatly in the oncology clinic. Thus, although we as oncologists may not be able to reduce drug prices or change the policies, there are actions that we can take in our clinic to reduce the costs of cancer treatment without necessarily compromising efficacy. Reducing financial toxicity at an individual patient level is seldom given priority, but this is important for three main reasons. First, it helps reduce financial toxicity to the patient and thereby improve health outcomes. Second, even if the patient is covered fully by insurance or able to afford it, we owe a collective responsibility to society to reduce the extra health care costs. Third, these actions will discourage the industry from developing me-too marginal drugs that provide low value to patients but incur a higher economic burden on health care systems.

Here, I point out a few examples of low value practices from various oncology disciplines (Table 1) that we oncologists can easily replace or abandon in our practice and contribute to lessening the financial toxicities to the patients and the society:

1. **Using ramucirumab in the second-line treatment of metastatic colorectal cancer:** After disease progression on first-line FOLFOX + bevacizumab therapy for metastatic colorectal cancer, FOLFIRI + bevacizumab should be preferred over the FOLFIRI + ramucirumab regimen as the second-line therapy [14]. Bevacizumab has evidence for efficacy in this setting (bevacizumab beyond progression) [15, 16] and is much cheaper than ramucirumab. The evidence for the use of ramucirumab has come from a trial where the comparator was placebo and not bevacizumab. These two angiogenesis inhibitors also share similar toxicity profiles. However, the cost of treatment per month for ramucirumab exceeds double that of bevacizumab. There is thus no good reason to use the expensive regimen of FOLFIRI + ramucirumab despite the availability of FOLFIRI + bevacizumab which has a similar strength of evidence, costs less and the long-term toxicities are better known. Both these drugs have not yet been compared against each other in the trials.
2. **Using anti-EGFR antibodies in the first-line treatment of right-sided metastatic colorectal cancer:** For RAS wild colorectal cancer patients, there are two choices of monoclonal antibodies for use with upfront chemotherapy: the anti-EGFR antibodies (cetuximab/panitumumab) or the angiogenesis inhibitor bevacizumab. Several retrospective analyses and meta-analyses have now shown that for right-sided tumours the anti-EGFR antibodies have very poor efficacy compared to bevacizumab [17, 19]. Based on these results, the NCCN guidelines have also now recommended that the anti-EGFR antibodies be used only for left-sided tumours [20]. However, not all oncologists are yet ready to change their practice. This is understandable given the lack of prospective data to support the claim that the anti-EGFR antibodies indeed perform poorly for right sided tumours. From the economics perspective, the anti-EGFR antibodies are very expensive compared to bevacizumab. For instance, cetuximab costs double per dose to that of bevacizumab. Also, studies have already demonstrated the cost-effectiveness of bevacizumab versus anti-EGFR antibodies in the treatment of colorectal cancer [21]. Now, what can possibly be the reason to keep prescribing cetuximab which is more expensive and possibly leads to poorer survival than bevacizumab for right-sided colon cancers? None. In any case, if presented with these data, probably no patient would choose cetuximab over bevacizumab in this setting irrespective of costs.
3. **Using cetuximab for concurrent use with radiotherapy in locally advanced head and neck squamous cell carcinoma (LAHNSCC):** Concomitant platinum-based chemoradiotherapy has been the standard of care in the management of LAHNSCC [22]. The 2006 Bonner trial established the role of cetuximab in combination with radiotherapy (RT) for similar setting [23]. However, this trial compared cetuximab-RT with RT alone, not against the standard treatment of platinum-based chemoradiotherapy. There are no trials comparing these two options head to head. On the other hand, panitumumab, also an anti-EGFR antibody like cetuximab, not only failed to show similar benefit in the recent CONCERT-1 and CONCERT-2 trials [24, 25] but showed inferior results. Cetuximab has just one phase-III data in its favour as opposed to numerous trials supporting platinum-based chemotherapy. Furthermore, cetuximab is extremely expensive compared to platinum agents. So, what good reasons do we have to use expensive cetuximab-RT which has a single RCT-based evidence instead of cheaper platinum-RT which has multiple meta-analyses-based evidence in the management of LAHNSCC? None; except for patients where cisplatin is contraindicated due to reasons like renal failure.
4. **Using single agent ramucirumab for second-line gastric cancer:** Ramucirumab as a single agent in the second-line treatment of gastric cancer was tested against placebo and not against the standard treatment of taxanes or irinotecan in the REGARD trial [26]. Even then, the drug marginally improved survival over *placebo* by 1.4 months. The median survival among patients that took ramucirumab was 5.2 months. While guidelines recommend that patients with an expected survival of less than 6 months should not be offered any invasive treatment, is there any value in prescribing this $13,000 per month drug that provides a survival of only 5.2 months? It should be remembered that patients receiving only placebo also had 3.8 months of survival and in a different trial, patients receiving standard chemotherapies such as paclitaxel or irinotecan have shown a survival of more than 8 months in the same setting [27]. Due to obvious pitfalls associated with cross-trial comparisons, this is not meant to imply that paclitaxel or irinotecan might have a better survival than ramucirumab but that other equally valid but cheaper options exist. While ramucirumab plus paclitaxel is a valid option in this setting, many patients are unable to tolerate this doublet therapy in a second-line setting.
5. **Using G-CSF for the treatment of febrile neutropenia (FN) in non-high-risk patients:** Although ASCO advocated against the rampant use of granulocyte-colony-stimulating factors (G-CSFs) for primary prophylaxis in patients at < 20% risk of developing FN as a part of the Choosing Wwisely initiative [28], it is no secret that the unwise use of G-CSFs is not limited to prophylaxis alone: equally rampant is the use of CSFs in the treatment of FN despite the lack of benefit. No guidelines (ASCO, NCCN, ESMO) suggest using CSFs for *treatment* of FN, except in high-risk patients. A 2014 Cochrane meta-analysis has also found that use of CSFs for the treatment of chemotherapy induced FN had a shorter duration of neutropenia (by a mean of 1.7 days) but no effect on infection-related or overall mortality [29]. Notwithstanding these recommendations, using CSFs has been a knee-jerk reaction to FN in oncology practice in many institutions. Thousands of dollars wasted for not-evidence based, not-recommended practice.
6. **Using chemotherapy towards the end of life:** The futility of using chemotherapy in patients with estimated survival of less than 6 months, irrespective of the performance status of the patient, has already been demonstrated [30]. Studies have also shown that oncologists tend to use anti-cancer drugs until the very end of life, despite the lack of benefit [31]. Although withholding anticancer treatment is a very delicate issue and we always prefer to err on the side of doing, we should understand that using chemotherapy in a dying patient is futile, in terms of both expenses and quality of life. The patient and the patient’s families need appropriate counselling. Sometimes, it is much easier to keep prescribing treatment than to sit with the patient and have a realistic conversation. But drugs are not the only thing a physician is expected to provide counselling, supportive care, and shared decision-making are other important but sometimes overlooked responsibilities of the physician. Irrespective of financial toxicity, no patient deserves to be under chemotherapy in the final days of his life. It is always an excellent idea to provide 60 minutes to the patient to discuss the end-of-life care rather than to prescribe chemotherapies in the last 60 days of his/her life.
7. **Testing CA-125 tests and CT scans for surveillance in ovarian cancer:** A well-conducted randomised controlled trial has previously shown that routine CA-125 tests for surveillance in ovarian cancer patients provides no survival benefit but increases chemotherapy use and worsens quality of life [32]. Another recent study has shown that physicians have continued to order these tests even after the publication of the RCT that showed lack of survival benefit with early institution of chemotherapy [33]. The surveillance cost is not trivial: for the US population, it was estimated to be $1,999,029 per year for CA-125 tests alone and $16,194,647 per year with CT scans added. An accompanying editorial questions ‘*But why would clinicians present the option of CA-125 testing? Shared decision making does not require that physicians present the patient with harmful options. We do not discuss heart transplants with patients who have mild congestive heart failure. Why would we discuss CA-125 testing with women who have ovarian cancer in remission?*’ and calls this ‘The Fatal Attraction of Testing’ [34]. Indeed, as other authors have shown, deimplementing medical practices are difficult and challenging [35]. However, if we are serious about addressing the financial toxicity of cancer treatment, deimplementing costly but ineffective practices are the first steps.
8. **Using sunitinib for the adjuvant treatment of renal cell carcinoma:** Although the S-TRAC trial has shown a benefit in disease-free survival with 1 year of adjuvant sunitinib [36], a well-powered previous trial had failed to show similar benefit [37]. In both the trials, no difference in overall survival has been reported yet. A meta-analysis of these two trials has shown no benefit in both disease-free and overall survival but increase in various toxicities [38]. Considering that a 1-year course of sunitinib costs more than $60,000 with no evidence of benefit in survival and possibly no benefit in disease free survival as well, what value does it offer?
9. **Ignoring cheaper drugs in supportive care:** Most of the discussions on financial toxicity of cancer treatment focus on expensive cancer drugs. However, drugs used for supportive care of cancer patients such as antiemetics for chemotherapy induced nausea and vomiting also add significantly to the overall cost, not only because they are expensive but also because they are used with every cycle of treatment. For example, using an olanzapine–palonosetron–dexamethasone regimen instead of the standard aprepitant–palonosteron–dexamethasone regimen provides similar protection against chemotherapy induced nausea and vomiting but costs nearly $500 less per cycle [39]. Similar other cheaper, evidence based alternatives should be sought, studied and implemented [39].

The examples offered here are not exhaustive. These are just a few low-value practices from various oncology disciplines that the author has personally come across. These practices are meant to provide stimulus and encourage discussions involving identifying and correcting various other low value practices in oncology clinics. The main intent of this paper is not to promote these specific practices but to motivate all oncologists to look for other similar strategies applicable to their own daily practice that can help potentially reduce the high expenses of anti-cancer treatment. Although the Choosing Wisely initiatives in various countries [28, 40] also focus on identifying low-value practices, they are more general recommendations and do not include guidance on specific treatment regimens or cancer drugs. The recommendations from the Choosing Wisely campaigns are well thought out and should definitely be implemented, but we should also keep looking for specific treatment avenues where low-value practices exist and can be corrected. The scenarios presented in this paper provide examples of real-life specific treatment decisions where low value practices can be corrected.

This consciousness of financial toxicity and opportunity to substitute low-value practices with better high-value alternatives is particularly important in countries where the national health insurance system bears all or most of the expenses. When the patient does not need to pay much (or at all) from their own pocket, both the physicians and patients tend to forget the issue of the high cost of therapies. This motivates both the physicians and patients to consider or choose expensive therapies with questionable or only marginal benefit. Therefore, it is important to educate both physicians and patients to consider the value of treatment in making decisions, especially in public health care systems. Although the government pays for the treatment, the government pays it through the money collected from the people and if expensive therapies are used rampantly, that share will have to be borne by all citizens.

Although some serious concerns have been raised lately regarding the impact of the financial toxicity of cancer treatment on society as a whole, most of the proposed solutions have focused on ways to reduce the skyrocketing cost of cancer drugs by government control or better academia-industry collaborations [41]. However, discouraging low-value practices in oncology clinics remains another important but often overlooked measure that can help reduce financial toxicity not only to the individual patient, but also to the entire health care system. It also indirectly helps reduce the price of cancer drugs by discouraging marginal me-too drugs.

## Conclusions

In conclusion, although we oncologists are limited in our capacities to fight against the increasing cost of cancer treatment, there are certain opportunities in our practice that we can change to reduce the overall financial toxicity. This paper provides a few examples of such low value practices that we can easily abandon or replace with better-value alternatives. As these examples suggest, reducing cost does not necessarily mean compromising efficacy. We should continuously keep looking for other similar cost-saving strategies in our practice. Identifying and implementing these cost-saving strategies will make a big difference in reducing the financial toxicity to our patients and societies.

## Conflicts of Interest

None

## Figures and Tables

**Table 1.** Examples of Low-value practices in oncology contributing to financial toxicity.

1. Using ramucirumab in the second-line treatment of metastatic colorectal cancer
2. Using anti-EGFR antibodies in the first-line treatment of right-sided metastatic colorectal cancer
3. Using cetuximab for concurrent use with radiotherapy in locally advanced head and neck squamous cell carcinoma
4. Using single agent ramucirumab for second-line gastric cancer
5. Using G-CSF for the treatment of febrile neutropenia in non-high-risk patients
6. Using chemotherapy towards the end of life
7. Testing CA-125 tests and CT scans for surveillance in ovarian cancer
8. Using sunitinib for the adjuvant treatment of renal cell carcinoma
9. Ignoring cheaper drugs in supportive care
